# Endoscopists' Acceptance on the Implementation of Artificial Intelligence in Gastrointestinal Endoscopy: Development and Case Analysis of a Scale

**DOI:** 10.3389/fmed.2022.760634

**Published:** 2022-04-12

**Authors:** Li Tian, Zinan Zhang, Yu Long, Anliu Tang, Minzi Deng, Xiuyan Long, Ning Fang, Xiaoyu Yu, Xixian Ruan, Jianing Qiu, Xiaoyan Wang, Haijun Deng

**Affiliations:** ^1^Department of Gastroenterology, The Third Xiangya Hospital of Central South University, Changsha, China; ^2^Health Management Center, The Third Xiangya Hospital of Central South University, Changsha, China; ^3^Department of Public Health, Yangtze University, Jingzhou, China

**Keywords:** artificial intelligence, endoscopist, scale, gastrointestinal endoscopy, analysis

## Abstract

**Background:**

The purpose of this paper is to develop and validate a standardized endoscopist acceptance scale for the implementation of artificial intelligence (AI) in gastrointestinal endoscopy.

**Methods:**

After investigating endoscopists who have previously used AI and consulting with AI experts, we developed a provisional scale to measure the acceptance of AI as used in gastrointestinal endoscopy that was then distributed to a sample of endoscopists who have used AI. After analyzing the feedback data collected on the provisional scale, we developed a new formal scale with four factors. Cronbach's alpha, confirmatory factor analysis (CFA), content validity, and related validity were conducted to test the reliability and validity of the formal scale. We also constructed a receiver operating characteristic (ROC) curve in order to determine the scale's ability to distinguish higher acceptance and satisfaction.

**Results:**

A total of 210 valid formal scale data points were collected. The overall Cronbach's alpha was 0.904. All the factor loadings were >0.50, of which the highest factor loading was 0.86 and the lowest was 0.54 (AVE = 0.580, CR = 0.953). The correlation coefficient between the total score of the scale and the satisfaction score was 0.876, and the area under the ROC curve was 0.949 ± 0.031. Endoscopists with a score higher than 50 tend to be accepting and satisfied with AI.

**Conclusion:**

This study yielded a viable questionnaire to measure the acceptance among endoscopists of the implementation of AI in gastroenterology.

## Introduction

In the field of gastroenterology, physicians need to process a large amount of clinical data and master various imaging devices as well (1). Taking esophagogastroduodenoscopy (EGD) as an example, procedural competence is a prerequisite for discovering lesions in EGD (2). A large number of guidelines and expert consensus have been reached to optimize EGD examination (3), but nonetheless, some studies have found that due to the significant differences in endoscopist performance in EGD, the discovery rate of discovery of gastric cancers (GCs) and precursor lesions is impaired (4). The diagnosis rate of early-stage GCs in China is still under 20%, and similar results are seen in most other parts of the world as well (5, 6).

Fortunately, the development of artificial intelligence (AI) can help solve the problem of misdiagnosis or missed lesions due to user error, especially for junior endoscopists. Previous studies have shown that AI can interpret specific medical images faster and better than humans (7), especially in detecting tiny polyps (8–10). Recently, the real-time quality improvement system WISENSE constructed based on deep reinforcement learning (DRL) and deep convolutional neural network (DCNN), has been shown to track suspicious cancerous lesions proactively and monitor blind spots, which can improve the quality of everyday endoscopy (11–13).

Although existing research shows that AI can help endoscopists perform endoscopic procedures better, the unilateral development of AI systems itself ignores the needs and expectations of endoscopists, who may be the most important stakeholders. With the development of AI, endoscopists may face new challenges and difficulties. For example, there are questions of whether the use of AI for diagnosis will make it difficult to identify cases of medical negligence, whether AI will affect the professional development of endoscopists, and whether using AI will make endoscopists dependent on it. Endoscopist preferences determine the boundaries within which an AI system functions. At present, however, little is known about endoscopists' acceptance of the use of AI in gastroenterology. In addition, there are no validated standardized questionnaires available for mapping endoscopists' acceptance of the implementation of AI in gastroenterology. This study therefore develops and validates a standardized endoscopist scale of acceptance on the implementation of AI in gastroenterology by means of expert evaluation, qualitative pretests, and factor analysis.

## Methods

Ethical approval for this study was provided by the Ethical Committee of the Third Xiangya Hospital of Central South University, China (permission received on December 31, 2019, No. 2019-S558).

### Provisional Scale Development

To develop our scale, we distributed questionnaires to endoscopists who have mainly used ENDOANGEL (11) (similar to the WISENSE system). The questionnaire contained some AI-related items regarding its use in other medical fields from previous research and the positive and negative affect schedule (PANAS) (14–16). Input on the questionnaire design was provided by the investigation and clinical medicine specialists from Central South University, China. We included a total of 29 questions that consisted of single-choice, multiple-choice, and open-ended questions pertaining to the ethics, emotions, effects, and training related to AI.

Based on this investigation, we identified four key factors of endoscopists' acceptance on AI implementation in endoscopy were identified: ethics, psychology and emotion, training, and accuracy. We used these four factors as an updated framework for the provisional scale and developed 5-7 items in each factor. We developed a total of 24 items, using five-point Likert-type agree-disagree scales. Seven demographic questions (birth date, gender, professional title, and information on the use of AI) were also included.

### Formal Scale Development

We distributed the original scale online by means of a QR code for endoscopists from three endoscopy centers in China where they use AI in gastrointestinal endoscopy. The data for a total of 42 questionnaires were collected from January 1, 2020 to January 5, 2020, and the overall Cronbach's alpha was 0.832. The factor loading for each of our 13 items was <0.50. Based on the results of the provisional scale questionnaire, the following modifications were made to the scale.

First, based on the use of AI in the Chinese endoscopy center, our four key factors were revised to consist of service, psychology and emotion, dependence, and accuracy. Some items were also deleted because they did not match the actual situation at the center, and some items were added that were related to the new key factors. Second, terminology was adjusted where it was sometimes interpreted as too general. Finally, the direction of all items was adjusted to provide positive verbiage regarding the AI, that is, toward stating that it was beneficial to the endoscopists to use AI. In this version, a total of 15 items, using five-point Likert-type agree-disagree scales, were developed. This version was then distributed online as a preliminary investigation for endoscopists from another three endoscopy centers in China, and the data were collected from January 14, 2020 to March 15, 2020. A total of 50 valid questionnaire scales were collected, and the overall Cronbach's alpha was 0.854. This time, however, the factor loading of all items was higher than 0.50. Therefore, we define this version of the scale as the formal scale.

### Formal Scale Data Collection

The endoscopists for formal scale data collection were recruited from April 1, 2020 until June 4, 2020. All the participants were from 10 endoscopy centers in China where AI is in use; none had been investigated before. Participation in the survey was voluntary and anonymous. Those who were reluctant to fill out the questionnaire and those who had never used AI were excluded. In addition, seven demographic questions were included in the survey, and some endoscopists were randomly selected to score their satisfaction with AI use (scoring 1-10 points, 10 points representing the most satisfaction).

### English Translation of the Formal Scale

The language of the formal scale sent to endoscopists was Chinese. Double translation was performed during the writing of this scale. A translator translated the scale into English and then sent it to an English-speaking foreign student who is proficient in Chinese. The student translated the English version of the scale back into Chinese again. A third translator then compared the two Chinese versions of the scale and polished the English version of the scale. Then, the revised English version was sent back to the foreign student who translated it into Chinese. The newly revised Chinese version was compared with the original version by the third translator, and he affirmed that the meaning of these two versions was the same.

### Statistical Analysis

The recorded data were analyzed by using IBM SPSS version 23.0 (IBM Corporation, Armonk, NY, USA), and Cronbach's alpha was used to calculate the internal consistency of items within each factor. In general, a Cronbach's alpha of 0.7 is taken as an indication of good internal consistency (17). The content validity was determined by expert evaluation and scoring, which was used to ensure that the respondents understood and answered the items in accordance with the content about which the item designer wished to ask. The maximum score of content validity was 10.

Confirmatory factor analysis (CFA) was performed to evaluate the correspondence between factors and items, and the loading of the first indicator in each factor was automatically fixed at 1.0. Multiple indices for fitness were used with the following criteria: root mean squared error of approximation (RMSEA) must be <0.08, with 90% confidence interval values below 0.10, and the Tucker-Lewis index (TLI) and the comparative fit index (CFI) must be >0.90 (18). We also analyzed the average variance extracted (AVE) and construct reliability (CR) and conducted a Pearson test was conducted in order to find the correlation between the total score of the scale and the satisfaction score (related validity). We used a satisfaction score ≥ 7 points (satisfied or very satisfied) as the state variable to draw the ROC curve. Based on this cut-off value, all respondents were divided into a high acceptance group and a low acceptance group. Finally, we performed a Chi-square test in order to explore the difference between the two groups based on demographic information.

## Results

### Sample

We collected a total of 166 scales the investigation using the formal scale. Since six respondents were excluded because they had not used AI before, we were left with 160 usable scales, with a valid rate of 96.39%. Since the formal scale and provisional scale quantitative properties were the same, and since the respondents were from different centers, we also included the sample from the provisional investigation in the reliability and validity analysis. From the provisional investigation we collected a total of 52 scales, but two respondents were excluded because they had never used AI before. This left us with 50 valid scales, with a validity rate of 96.15%. Hence, we had a total of 218 scales and 210 valid scales between the two investigations, with a total validity rate of 96.33%. In addition, 40 scales included satisfaction with AI. The demographic information is shown in Table 1.

**Table 1:** The demographic information of all respondents.

	**Options**	***N***
Sex	Male	122 (58.10%)
	Female	88 (41.90%)
Age	Under 17 years	0 (0.00%)
	18-25 years	2 (0.95%)
	26-40 years	135 (64.29%)
	41-65 years	72 (34.29%)
	Over 66 years	1 (0.48%)
Rank of the hospital	First-class hospital	176 (83.81%)
	Second-class hospital	32 (15.24%)
	Third-class or lower hospital	2 (0.95%)
Professional title	Chief physician or associate chief physician	96 (45.71%)
	Physician-in-charge	92 (43.81%)
	Physician	22 (10.48%)
Whether used AI	Yes	210 (100.00%)
	No	0 (0.00%)
The duration of using AI	<1 month	25 (11.90%)
	1-3 months	36 (17.14%)
	3-6 months	60 (28.57%)
	6 months to 1 year	37 (17.62%)
	Over 1 year	52 (24.76%)
On how many patients have you performed endoscopy with AI?	<50 patients	43 (20.48%)
	50-100 patients	55 (26.19%)
	100-300 patients	55 (26.19%)
	300-500 patients	25 (11.90%)
	Over 500 patients	32 (15.24%)

### Results of Reliability and Validity Tests

**Figure 1:**
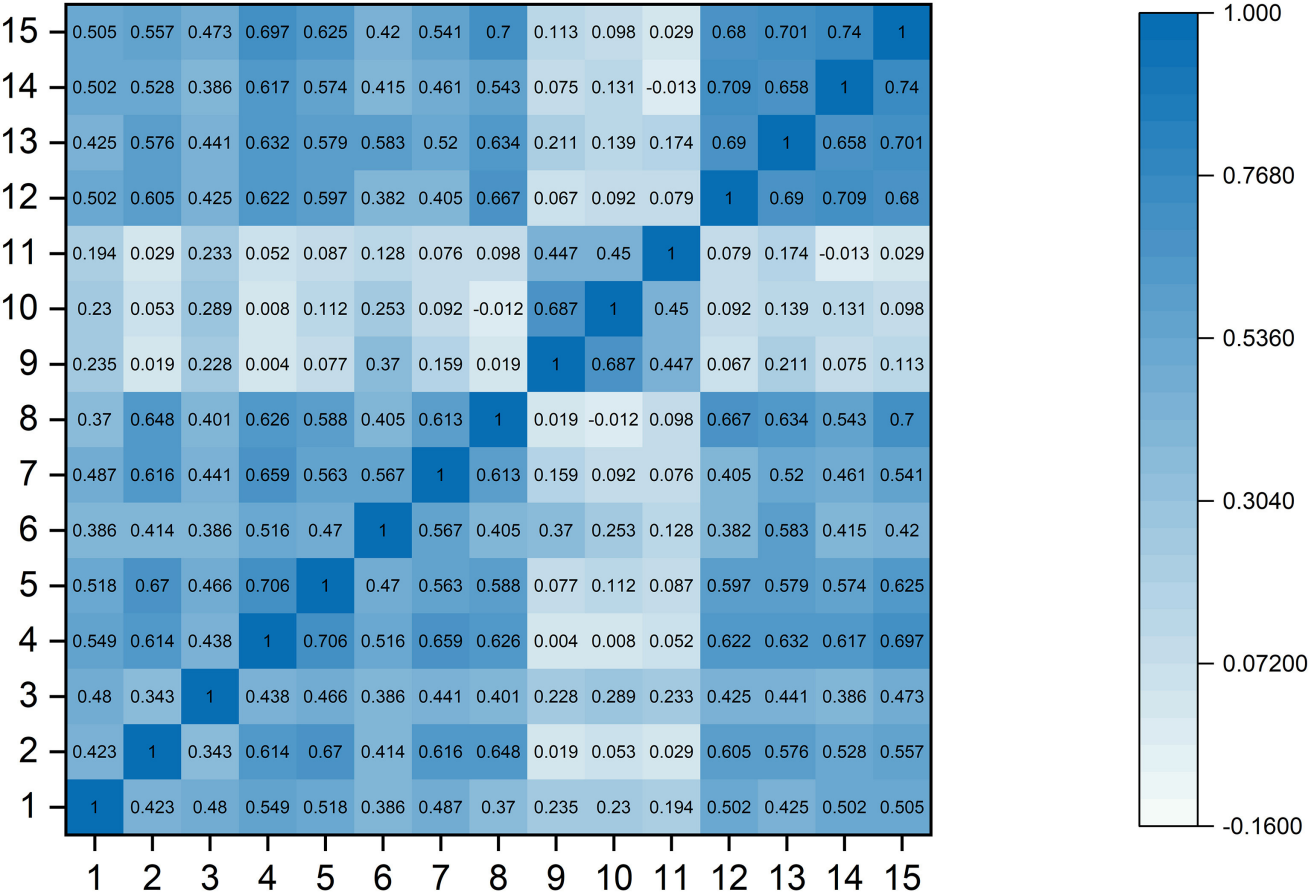
The correlation matrix of all 15 items.

**Figure 2:**
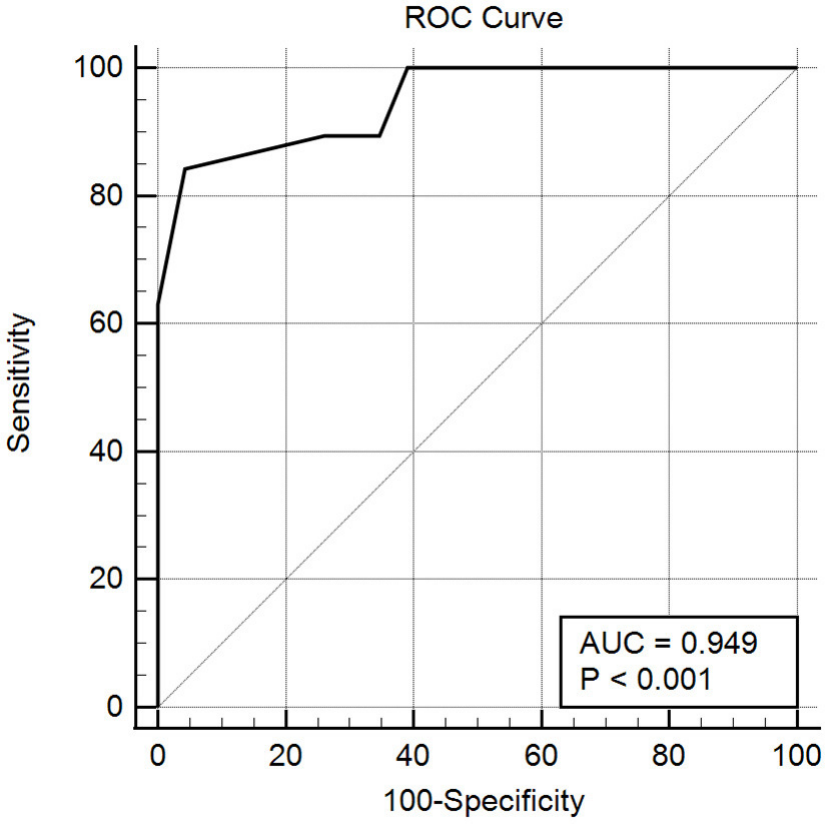
The ROC curve of the total scores of the scale measuring whether the endoscopists were satisfied with AI. The AUC of the curve was 0.949 ± 0.031. Endoscopists with a score higher than 50 tended to be accepting and satisfied with AI (the Youden index was 0.799 with 84.21% sensitivity and 95.65% specificity).

Five experts participated in our content validity rating, and the average score was 9. The overall Cronbach's alpha was 0.904, and the Cronbach's alpha of the four factors was 0.778, 0.828, 0.773, and 0.901, respectively. All the factor loadings were >0.50 (Table 2, AVE = 0.580, CR = 0.953). The RMSEA was 0.079, the TLI was 0.901, and the CFI was 0.905. Additionally, the correlation matrix of all 15 items showed that most of the pairwise correlations between items (96.19%) were <0.7 (Figure 1). The correlation between these four factors was also tested (Table 3), and the Pearson test showed that the correlation coefficient between the total score of the scale and the satisfaction score was 0.876 (*P* < 0.001). Importantly, the ROC curve demonstrated that the total scores of the scale could efficiently differentiate whether endoscopists were satisfied with AI, with an area under the curve (AUC) of 0.949 [95% CI: 0.833-0.993] (Figure 2).

**Table 2:** The descriptive figures of 15 attitudinal items for each of the 4 factors of the scale.

**Item**	**Mean**	**Standard deviation**	**Factor loading**
Overall, Cronbach's alpha 0.904	3.76	0.279	-
**Factor 1. Service**			
Overall Cronbach's alpha 0.778	3.66	0.329	-
1. The endoscopists would be less responsible for medical negligence when using the AI.	3.18	1.399	0.64
2. Using the AI will increase the patient's confidence in the endoscopists' diagnosis	3.89	1.004	0.76
3. The widespread popularity and development of AI will not adversely affect the employment and promotion of endoscopists.	3.86	1.069	0.58
4. Using AI will reduce the workloadof the endoscopist.	3.69	1.009	0.82
**Factor 2. Psychology and emotion**			
Overall Cronbach's alpha 0.828	3.81	0.179	-
5. Using AI will bring psychological comfort to the endoscopists.	3.62	1.105	0.86
6. Using AI will not let the endoscopists neglect to improve the performance of endoscopy.	3.71	1.204	0.60
7. Using AI will make the endoscopists more interested in performing endoscopy.	3.87	0.906	0.75
8. Using AI will make the endoscopistsconcentrate more on the endoscopy procedure.	4.03	0.904	0.78
**Factor 3. Dependence**			
Overall Cronbach's alpha 0.773	3.45	0.110	-
9. Endoscopists who are used to AI will not miss any blind spots even if they do not use AI.	3.48	0.999	0.80
10. Endoscopists who are used to AI will not miss any lesions even if they do not use AI.	3.33	1.031	0.85
11. For endoscopists who are used to AI, even if they do not currently use AI, their withdrawal time will not be extended.	3.54	0.875	0.54
**Factor 4. Accuracy**			
Overall Cronbach's alpha 0.901	4.03	0.063	-
12. AI can improve the accuracy of the diagnosis of lesions.	4.12	0.827	0.82
13. AI can improve the sensitivity of the diagnosis oflesions.	4.03	0.933	0.82
14. AI can improve the specificity of the diagnosis of lesions.	4.01	0.897	0.82
15. AI can accurately identify blind spots.	3.97	0.909	0.87

**Table 3:** Correlations between factors.

	**Factor 1**	**Factor 2**	**Factor 3**	**Factor 4**
Factor 1	-			
Factor 2	0.97	-		
Factor 3	0.21	0.11	-	
Factor 4	0.88	0.90	0.16	-

### Endoscopists' Acceptance of AI in Gastroenterology

The average total score of the scale was 56.34, and the median total score of the scale was 54. Item 1 scored lowest on the whole scale, with an average score of 3.18. Compared with the other three factors, factor 3 (dependence) had the lowest average score, at 3.45. Endoscopists rated item 10 the lowest, with an average score of 3.33.

Based on the cut-off value, a total of 167 (79.52%) endoscopists had high acceptable and were satisfied with AI (Table 4). There was significant difference between high and low acceptability in age (*P* = 0.013), professional title (*P* = 0.001), and the duration of using (*P* = 0.000) AI. For professional title, there was significant difference between physician and chief physician or associate chief physician (*P* = 0.001) or physician-in-charge (*P* = 0.001). Compared with the other durations of using ENDONGEL, respondents who use the novel AI system <1 month were more likely to have low acceptance (*P* = 0.008, 0.006, 0.001, 0.000).

**Table 4:** The acceptance of all respondents based on demographic information.

**Feature**	**High acceptance (*N* = 167, 79.52%)**	**Low acceptance (*N* = 43, 20.48%)**	***P***
**Sex**			
Male	97 (58.08%)	25 (58.14%)	0.995
Female	70 (41.92%)	18 (41.86%)	
**Age**			
≤ 40 years	102 (61.08%)	35 (81.40%)	0.013
>41 years	65 (38.92%)	8 (18.60%)	
**Rank of the hospital**			
First-class hospital	141 (84.43%)	35 (81.40%)	0.630
Second-class and Third-class hospital	26 (15.57%)	8 (18.60%)	
**Professional title**			
Chief physician or associate chief physician	79 (47.31%)	17 (39.53%)	0.001
Physician-in-charge	77 (46.10%)	15 (34.88%)	
Physician	11 (6.59%)	11 (25.58%)	
**The duration of using AI**			
<1 month	12 (7.19%)	13 (30.23%)	0.000
1-3 months	29 (17.37%)	7 (16.28%)	
3-6 months	47 (28.14%)	13 (30.23%)	
6 months to 1 year	33 (19.76%)	4 (9.30%)	
Over 1 year	46 (27.54%)	6 (13.95%)	
**On how many patients have you performed endoscopy with AI?**			
<50 patients	31 (18.56%)	12 (27.91%)	0.189
50-100 patients	42 (25.15%)	13 (30.23%)	
100-300 patients	43 (25.75%)	12 (27.91%)	
300-500 patients	21 (12.57%)	4 (9.30%)	
Over 500 patients	30 (17.96%)	2 (4.65%)	

## Discussion

To the best of our knowledge, this is the first paper in the literature to create a scale to measure investigate endoscopists' acceptance on the implementation of AI in gastrointestinal endoscopy. New developments in AI have advanced tremendously in recent years, and AI is expected to cause a new digital revolution in the coming decades (19). Many scholars believe that physicians will use AI technology, particularly deep learning, in the future (7), and researchers anticipate that gastrointestinal endoscopy is one of the fields that will be transformed significantly.

Many studies have suggested that AI especially can assist endoscopists in many aspects (7–9, 11–13). In particular, displaying the examined site, reducing the blind spot rate of endoscopy and diagnosing the lesions are commonly used by endoscopists. However, there is a lack of debate on how endoscopists would perceive such a transformation. The development of AI may make endoscopists face other problems and challenges, such as ethical issues and psychological and emotional changes. Therefore, we investigated the opinions of endoscopists on AI directly, and our results may help endoscopic centers design their deployment of AI. More importantly, it can help AI improve the areas that endoscopists generally think are inappropriate, so that AI and endoscopists can be more compatible. For example, we found that endoscopists have negative views of dependence, so the development of AI needs to consider reducing endoscopists' dependence on AI. In addition, the development of the scale for endoscopists also provided inspiration for the development of a scale for patients in the future.

In this study, we documented the development of a standardized scale to measure endoscopists' attitudes toward AI in gastroenterology. Due to the lack of an existing standard scale, we cannot conduct criterion-related validity. In order to make up for this shortcoming, 40 endoscopists were asked about their satisfaction with the artificial intelligence. Based on this, the correlation test and receiver operating characteristic (ROC) curve were conducted, and the results showed that a score of 50 points can be used as a critical value to differentiate whether the endoscopist was satisfied with AI. In addition, the correlation coefficient between the total score of the scale and the satisfaction score was 0.876. These results indicate that our scale had good reliability as well as validity.

According to the results of our investigation, Chinese endoscopists were optimistic about AI in general. However, we discern see the endoscopists' concerns about AI from their responses to some items. For example, when using AI resulted in medical negligence, such as a misdiagnosis, the question of who would be responsible was the most worrying to endoscopists and produced the most divergent opinions among them. In fact, the ethical and legal issues of AI are currently the subject of intense debate in other medical fields, such as radiology (20, 21). However, only one very relevant item was retained. The current development of AI in China is still in its nascent stage. The patient or the endoscopist might not understand AI, and the understanding of ethical and legal issues is not enough. Therefore, we set ethics as a factor in the original version of the scale, but this factor was deleted in the formal version of the scale due to the unsatisfactory results of the investigation. Similar to the above reason, the factor of using AI for endoscopic training was also deleted in the formal scale.

The problem of using AI to generate dependencies has never been explored in previous research, but this may indeed become an important issue due to the popularity of AI. Judging from the results of our investigation, endoscopists were very optimistic about this technology. However, most of the endoscopists currently using AI were senior physicians, which might bias the results. Especially for beginners who use AI or similar systems for training, there is an urgent need to determine whether the endoscopy procedure can be performed and whether the lesions can be diagnosed without AI.

Previous studies have found that AI has a high accuracy, sensitivity, and specificity in identifying lesions and blind spots (12, 13), which is similar to the results of our formal-scale investigation. However, we found that despite the findings of the above studies, there were still endoscopists who did not trust the diagnoses of AI. This may cause a misdiagnosis when endoscopists ignores the suggestion of the AI because they distrust its accuracy, and this situation is contrary to the purpose of developing AI to assist endoscopists in diagnosis. Therefore, we designed items about AI accuracy in the formal version of the scale. In addition, if the endoscopist is optimistic about the accuracy of AI (factor 4), then it is likely to have a positive effect on the endoscopists feelings regarding the services provided by AI (factor 1) and the endoscopist's psychological and emotional changes (factor 2). However, if endoscopists are optimistic about factor 1 or 2, they may also be optimistic about factor 4. This may explain why these three factors are highly correlated.

We also analyzed high and low acceptance based on the demographic information of all respondents. We found that age, professional title, and duration of using AI were three aspects where significant differences were observed. Older endoscopists and those with higher professional titles were more likely to be satisfied with the AI system. This is different from what we presumed at the beginning, since AI as a novel system may be more acceptable to young people, and further study may be needed.

Finally, this article has several limitations. First, most of our respondents were from first-class hospitals, and their acceptance of AI may be different from those of endoscopists from primary hospitals. Additionally, since many AIs systems have only recently become a medical product marketed in China, after AI is more widely popular in China, the existing items on our formal scale may not fully measure endoscopists' acceptance of AI. Due to these limitations, we intend to overcome these shortcomings in our follow-up research so that our scale can be better used by researchers and endoscopy centers.

## Conclusion

In conclusion, our study yielded a viable questionnaire to measure acceptance among endoscopists regarding the implementation of AI in gastroenterology. We find that endoscopists with a score higher than 50 had a higher acceptance and satisfaction with AI.

## Data Availability Statement

The original contributions presented in the study are included in the article/supplementary material, further inquiries can be directed to the corresponding author/s.

## Ethics Statement

The studies involving human participants were reviewed and approved by Ethics Committee of Xiangya Third Hospital of Central South University, Changsha, Hunan Province. The patients/participants provided their written informed consent to participate in this study.

## Author Contributions

LT and ZZ were responsible for study concept and design, acquisition of data, analysis and interpretation of data, statistical analysis, and drafting of the manuscript. YL, AT, MD, XL, and NF were responsible for acquisition of data, analysis and interpretation of data, and critical revision of the manuscript for important intellectual content. XY and XR were responsible for acquisition of data and critical revision of the manuscript for important intellectual content. XW and HD oversaw collection and analysis of the data and had final responsibility for the decision to submit for publication. All authors had full access to all the data in the study and reviewed and approved the final manuscript.

## Conflict of Interest

The authors declare that the research was conducted in the absence of any commercial or financial relationships that could be construed as a potential conflict of interest.

## Publisher's Note

All claims expressed in this article are solely those of the authors and do not necessarily represent those of their affiliated organizations, or those of the publisher, the editors and the reviewers. Any product that may be evaluated in this article, or claim that may be made by its manufacturer, is not guaranteed or endorsed by the publisher.

## Abbreviations

AI: artificial intelligence
CFA: confirmatory factor analysis
EGD: esophagogastroduodenoscopy
GCs: gastric cancers
DRL: deep reinforcement learning
DCNN: deep convolutional neural network
PANAS: positive and negative affect schedule
RMSEA: root mean squared error of approximation
TLI: Tucker-Lewis index
CFI: comparative fit index
AVE: average variance extracted
CR: construct reliability
ROC: receiver operating characteristic.
